# Hospitalizations due to primary care-sensitive conditions among children under five years of age: cross-sectional study: Internações por condições sensíveis à atenção primária em crianças menores de cinco anos: estudo transversal

**DOI:** 10.1590/1516-3180.2016.0344250217

**Published:** 2017-04-03

**Authors:** Erika Morganna Neves de Araujo, Gabriela Maria Cavalcanti Costa, Dixis Figueroa Pedraza

**Affiliations:** I BSc. Master’s Student, Postgraduate Program on Public Health, Universidade Estadual da Paraíba (UEPB), Campina Grande (PB), Brazil.; II PhD. Professor, Postgraduate Program on Public Health, Universidade Estadual da Paraíba (UEPB), Campina Grande (PB), Brazil.

**Keywords:** Primary health care, Child care, Child health, Hospitalization, Length of stay

## Abstract

**CONTEXT AND OBJECTIVE:**

Hospitalizations due to primary care-sensitive conditions constitute an important indicator for monitoring the quality of primary healthcare. This study aimed to describe hospitalizations due to primary care-sensitive conditions found among children under five years of age (according to their age and sex), in two cities in Paraíba, Brazil.

**DESIGN AND SETTING:**

Cross-sectional study carried out in the municipalities of Cabedelo and Bayeux, in Paraíba, Brazil.

**METHODS:**

Data were collected from four public pediatric hospitals in Paraíba that receive children from these municipalities. Hospital admission authorizations were consulted to gather information on the children’s profile and the characteristics of their hospitalizations. Differences in the causes of admissions and the respective lengths of hospital stay length were analyzed according to age group and sex.

**RESULTS:**

The proportion of hospital admissions due to primary care-sensitive conditions was 82.4%. The most frequent causes were: bacterial pneumonia (59.38%), infectious gastroenteritis and its complications (23.59%) and kidney and urinary tract infection (9.67%). Boys had higher frequency of hospitalizations due to primary care-sensitive conditions than girls. The median hospitalization due to primary care-sensitive conditions was found to be four days. The duration of hospital stays due to primary care-sensitive conditions was significantly longer than those due to conditions that were not sensitive to primary care.

**CONCLUSIONS:**

High rates of hospital admissions due to primary care-sensitive conditions were highlighted, especially among children of male sex, with long periods of hospitalization.

## RESUMO

**CONTEXTO E OBJETIVO:**

As internações por condições sensíveis à atenção primária constituem importante indicador para o monitoramento da qualidade da atenção primária à saúde. O presente estudo objetivou descrever as internações por condições sensíveis à atenção primária em crianças menores de cinco anos (por idade e sexo) em duas cidades da Paraíba.

**TIPO DE ESTUDO E LOCAL:**

Estudo transversal realizado nos municípios de Cabedelo e Bayeux, ­Paraíba, Brasil.

**MÉTODOS:**

Coletaram-se os dados nos quatro hospitais públicos pediátricos da Paraíba que internam crianças residentes nos municípios estudados. A partir das autorizações de internação hospitalar, colheram-se informações relativas ao perfil da criança e características das internações. Analisaram-se as diferenças nas causas de internações e respectivos tempos de hospitalização segundo faixa etária e sexo.

**RESULTADOS:**

A proporção de internação por condição sensível à atenção primária foi de 82,4%. As causas mais frequentes foram: pneumonias bacterianas (59,38%), gastroenterites infecciosas e suas complicações (23,59%) e infecção do rim e trato urinário (9,67%). Meninos apresentaram maior frequência de internações por condições sensíveis à atenção primária do que meninas. Verificou-se mediana de quatro dias de hospitalização para as condições sensíveis à atenção primária. O tempo de hospitalização por condição sensível à atenção primária foi significantemente maior do que o tempo da condição não sensível à atenção primária.

**CONCLUSÕES:**

Ressaltam-se altas taxas de internações por condições sensíveis à atenção primária, principalmente em crianças do sexo masculino, com longos períodos de hospitalização.

## INTRODUCTION

The indicator “hospitalizations due to primary care-sensitive conditions” was initially proposed in the United States in the 1990s to describe health conditions in which effective primary care provided at the right time can help to reduce or eliminate the need for hospital admissions, because prevention and proper treatment can be applied at early stages of the disease.^1^^,^^2^ Hospital admissions due to primary care-sensitive conditions have increasingly been used as quality indicators worldwide because of their importance for monitoring and evaluating primary healthcare.^2^

Hospitalizations due to primary care-sensitive conditions have been classified and listed for application in Brazil.^3^^,^^4^ When used as a performance indicator, hospital admissions due to primary care-sensitive conditions should be applied to age groups separately in order to better analyze primary healthcare quality.^3^

Despite the importance of hospital admissions due to ambulatory care-sensitive conditions, studies targeting children under five years of age in Brazil are few and concentrated in the southeastern region of this country.^2^ This indicator has great potential for evaluating the quality of primary healthcare for acute conditions that is provided for this age group. Such conditions lead to an associated high probability of hospital admission among these users.^3^^,^^5^ Children also present greater vulnerability to social determinants of health and to worsening of diseases than adults, thus making their healthcare a priority.^6^

## OBJECTIVE

To describe the hospitalizations due to primary care-sensitive conditions that were identified among children under five years of age (according to age and sex) who were admitted to pediatric hospitals in two cities in Paraíba, Brazil.

## METHODS

This was a cross-sectional study on hospital admissions of children under five years of age who were living in the municipalities of Cabedelo and Bayeux, Paraíba. These cities were chosen based on their similarities regarding characteristics such as geographical location (in the metropolitan region of the state capital with access to the network of contracted services), degree of urbanization, sociodemographic indicators, economic resources and tradition within the organization of primary healthcare services (the Family Health Strategy covers nearly 100% of the population). Cabedelo has a healthcare system composed of 28 Family Health Strategy teams and Bayeux, 19 teams.

The field team for the present study was composed of healthcare professionals and students with previous experience of fieldwork. The quality control for the study included training and standardizing the interviewers, preparing an instruction manual, carrying out a pilot study and supervising the data collection process.

Data were collected between March 12 and 21, 2014, from four pediatric public hospitals in Paraíba: one located in Cabedelo and three in João Pessoa. These hospitals are responsible for pediatric hospitalizations for families living in the municipalities studied. The medical and statistical record service of each institution granted permission for use of the hospital records relating to all children born between 2008 and 2013 who were hospitalized in 2013.

Firstly, all children living in the municipalities of interest for whom the difference between their date of birth and date of admission was less than five full years were identified through directly consulting the records. This resulted in the sample size of the study (n = 627).

Next, hospital admission authorizations were analyzed to identify the reasons for hospital admission. Admissions were classified as due to primary care-sensitive conditions or not. For this purpose, the Brazilian list of hospitalizations due to primary care-sensitive conditions was used as a reference.^7^ Data on the children’s characteristics (sex and birth date) and admissions (length of hospital stay and outcome) were also obtained. Each child’s age was calculated as the difference between the consultation date and the birth date, and was then classified into categories (< 25 months or 25-60 months).

The data were doubled-entered and organized in spreadsheets. The “Validate” application of the Epi Info software (version 3.3.2) was used to analyze data consistency, and then the final database that was used in statistical analyses was generated.

The frequency of each cause of hospital admission and its proportional contribution to the total number of admissions and the number of admissions due to primary care-sensitive conditions were assessed. The rates of admissions due to primary care-sensitive conditions in relation to the population living in each municipality in 2013 (for the total population and for children under five) were also analyzed.

The chi-square test was used to analyze differences between causes of admissions according to the children’s age group and sex. For children for whom the outcome was discharge, the length of stay (days) was expressed as the median and interquartile range. Differences in length of hospital stay according to age and sex were tested through the Mann-Whitney test. The same test was used for pairwise comparisons of length of hospital stay due to each ambulatory care-sensitive condition.

The normality of the data was checked using the Kolmogorov-Smirnov test and the significance level accepted was 5%, for all statistical analyses. We used the Statistical Package for the Social Sciences (SPSS) software, version 13.0, for the analyses.

This study was approved by the Ethics Committee of the State University of Paraíba, under protocol number 19689613.3.0000.5187.

## RESULTS

Among the 627 children under five years of age living in the municipalities of Bayeux and Cabedelo who were admitted to pediatric hospitals in the state of Paraíba in the year 2013, 55.2% were male and 52.6% were aged 25 months or over. The majority of these children were hospitalized for one to five days (73.1%) (Table 1).

**Table 1:**
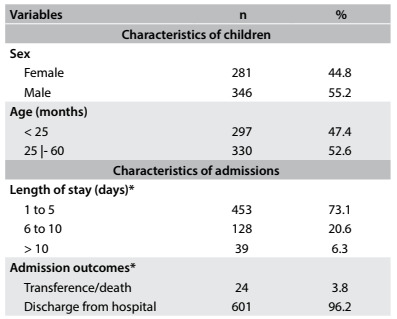
Characteristics and hospital admissions among children under five years of age. Cabedelo and Bayeux, Paraíba, 2013

*Values that differ from the total are due to lack of information on hospital admission orders.

Table 2 shows that 82.46% of the total number of admissions were due to primary care-sensitive conditions. Bacterial pneumonia (59.38%), infectious gastroenteritis and its complications (23.59%) and kidney and urinary tract infections (9.67%) were the main causes of admissions due to ambulatory care-sensitive conditions. These represented 76.39% of the total admissions and 95.92% of those due to ambulatory care-sensitive conditions. The frequencies of hospitalizations per specific cause ranged from 1.34/1000 (skin and subcutaneous tissue infections) to 24.16/1000 (bacterial pneumonia) in this sample of children under five years.

**Table 2:**
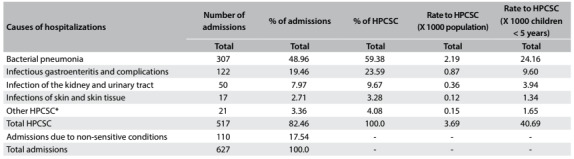
Causes of hospital admissions of children under five years of age: frequencies, proportions and hospitalization due to primary care-sensitive conditions (HPCSC) in Cabedelo and Bayeux, Paraíba, 2013

*Causes of admissions that presented low frequency: asthma; diseases of the lower airways; ear, nose and throat infections; whooping cough; epilepsy; malnutrition; anemia.

 Regarding age group (Table 3), it was observed that infectious gastroenteritis and its complications was more frequent among children between 25 and 60 months of age. Among children younger than 25 months, bacterial pneumonia predominated.

**Table 3:**
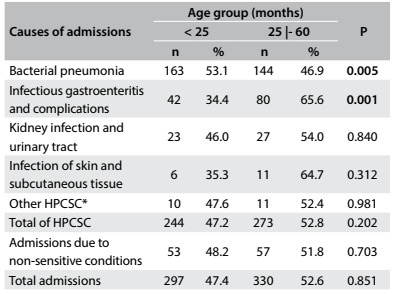
Causes of hospital admissions of children under five years of age according to age group. Cabedelo and Bayeux, Paraíba, 2013

*Causes of admissions that showed low frequencies = asthma; diseases of the lower airways; ear = nose and throat infections; whooping cough; epilepsy; malnutrition; anemia. P = values-P for the chi-square test; P in bold denotes statistical significance; HPCSC = hospitalizations due to primary care-sensitive conditions.

Boys were hospitalized more often (P = 0.017) for primary care-sensitive conditions such as infections gastroenteritis and skin conditions (P < 0.02) than girls. This was observed for infectious gastroenteritis and its complications, and for skin and subcutaneous tissue infections (Table 4).

**Table 4:**
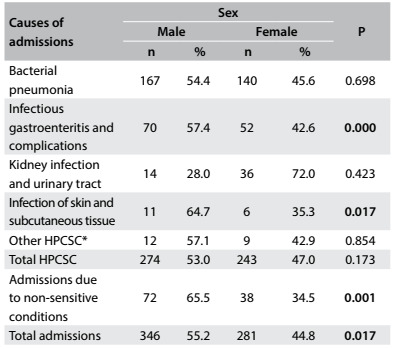
Causes of hospital admissions of children under five years of age according to sex. Cabedelo and Bayeux, Paraíba, 2013

*Causes of admissions that showed low frequencies: asthma; diseases of the lower airways; ear, nose and throat infections; whooping cough; epilepsy; malnutrition; anemia. P = P-values for the chi-square test; P in bold denotes statistical significance; HPCSC = hospitalizations due to primary care-sensitive conditions.

Children admitted due to primary care-sensitive conditions were hospitalized for longer times than children admitted due to conditions that were not sensitive to primary care. Among the primary care-sensitive causes, the length of hospital stay due to infectious gastroenteritis and its complications was significantly lower than that found for the other groups of causes. Considering all admissions, the length of hospital stay was longer for girls than for boys (Table 5).

**Table 5:**
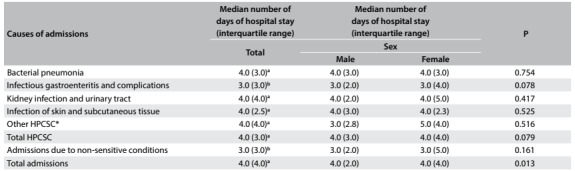
Hospital stay length of the causes of admissions of children under five years of age according to the child’s sex. Cabedelo and Bayeux, Paraíba, 2013

*Causes of admissions that showed low frequencies: asthma; diseases of the lower airways; ear, nose and throat infections; whooping cough; epilepsy; malnutrition; anemia. P = P-values for the chi-square test; P in bold denotes statistical significance; P > 0.05 for the analysis by age group; ^a,b^Different superscript letters denote statistical difference in length of hospital stay among the causes of hospitalizations; HPCSC = hospitalizations due to primary care-sensitive conditions.

## DISCUSSION

The present study showed that a high proportion of admissions were due to ambulatory care-sensitive conditions. Bacterial pneumonia, infectious gastroenteritis and its complications, kidney and urinary tract infection and skin and subcutaneous tissue infection presented the highest proportions. In other localities of Brazil, primary care-sensitive conditions were also found to be the main reason for hospitalization among children.^5^^,^^6^ Thus, the social, economic and cultural complexity of the reality and demands of this population group needs to be considered in healthcare planning.^8^^,^^9^

The high number of hospitalizations among the children in the present study was attributed to cases of bacterial pneumonia, infectious gastroenteritis and its complications, kidney and urinary tract infection and skin and subcutaneous tissue infection. This corroborates a previous study that also showed the same clinical conditions as the main causes of primary care-sensitive hospitalizations.^3^^,^^10^ Similar results have been also systematized recently.^2^ These acute conditions are highly prevalent among the sicknesses affecting children, whereas greater diversification and variability of illnesses are observed in the adult population as a consequence of aging and higher levels of poor lifestyle choices.^3^^,^^5^ The vulnerability to illness among children under five years of age is associated with accelerated physical growth and age-specific frailty.^11^

The two main primary care-sensitive causes observed in the study population, namely bacterial pneumonia and infectious gastroenteritis and its complications, have also been observed in other scenarios in Brazil.^3^^,^^9^^,^^12^ These diseases may be related to environmental and socioeconomic conditions, in particular water purification, waste water treatment and air quality. Thus, prevention needs to be strengthened through guidance for children’s caregivers and should cover integration between various sectors of public policy.^3^^,^^13^^,^^14^ Kidney and urinary tract infection ranked as the third largest cause of hospital admission among the children in this study, and this was similar to what was found in Paraná^3^ and was recently systematized.^2^ Importantly, it is possible to diagnose and treat these conditions at their early stages through urine analyses and pharmacological treatment with antibiotic drugs.^2^^,^^15^

The vulnerability of young children to hospitalization due to bacterial pneumonia that was observed in the current study was similar to the findings from a previous study.^3^^,^^13^ This can be explained by the immaturity of the immune system of younger children, which is associated with smaller-caliber airways that impose difficulty in the process of removing foreign elements.^11^ In the context of healthcare, higher numbers of admissions among children under one year of age is mostly associated with inadequate care during the prenatal period, at delivery and during the postpartum period.^3^

In a survey carried out in São José do Rio Preto, state of São Paulo,^9^ and in the current study, older children were more frequently hospitalized due to infectious gastroenteritis and its complications. Other studies have reported higher prevalence of admissions due to this cause among younger children than those in the current study.^1^^,^^3^^,^^16^ Considering the peculiarity of the immaturity of the immune system of young children, who have greater vulnerability to hydroelectrolytic disorders and bacterial and protozoan etiological agents,^1^^,^^3^^,^^16^ it is feasible that these rates may be influenced by greater care given to children younger than 25 months of age. Older children who are also in a vulnerable and immature physiological condition are exposed more often to degraded environments and to their own family’s socioeconomic situation.^3^

Previous studies carried out in Brazil identified higher prevalence rates of admissions among male children,^14^^,^^17^ as in the present report. Greater exposure to infectious agents, among Brazilian boys because of their greater freedom to participate in sports, for sociocultural reasons, probably leads to more hospitalizations of boys than girls.^16^

Length of hospital stay is an indicator of quality of care. Likewise, admissions due to primary care-sensitive conditions and length of stay can also be reduced by providing qualified care in primary healthcare services.^4^^,^^18^ In spite of this, we were unable to find any study addressing this indicator among Brazilian children, from this perspective. The average length of hospital stay found in this study was four days. The same was found in Colombia.^18^ A longer length of stay was reported in the city of Montes Claros, Minas Gerais.^5^ This amount of time can be considered long, since the causes of admissions can be treated with antibiotics, which are easily available and effective within 72 hours.^15^^,^^19^ Deficiencies in the pediatric hospital network of the municipalities studied possibly have a role in this result. Other authors have also found longer hospital stays among children who did not live in the state capital.^20^ This hypothesis is supported by the fact that infectious gastroenteritis and its complications required shorter hospital stays, most likely because of its low complexity and because of the possibility that this condition can be resolved within the home environment through simple measures such as rehydration, rest and a bland diet.^16^

A study conducted in the United States showed that hospital stays were longer among girls than among boys,^1^^,^^2^ just like in the present study. The explanations for sex-specific differences in length of hospital stay have been found in the scientific literature to be inconsistent and, thus, further investigation is merited.^21^ It is noteworthy that length of hospital stay is a complex variable that depends on factors such as clinical condition, severity of the disease and the possibility of complications.^18^^,^^19^

It is important to note that some determinants of admissions among children were not addressed in this study. These included socioeconomic conditions, which have clear associations with access to health services.^1^ Furthermore, families also have a role regarding adherence to the guidelines for child healthcare and placing value on the Family Health Strategy service, as a gateway to the healthcare system.^5^^,^^8^ The quality of healthcare and the profile of the professionals involved are other components of admissions due to primary care-sensitive conditions that were not considered here.^22^^,^^23^

The current study has other limitations that need to be discussed. The first relates to use of hospital admission authorizations for obtaining the reason for hospitalization. The purpose of this instrument is primarily to document the financial reimbursement for services rendered. Thus, there may be flaws in data registration in the hospital (which may or may not be intentional) that compromise the accuracy of the information. The second limitation relates to the focus on services financed through the public system alone, even though hospitalizations due to primary care-sensitive conditions may also occur within the private healthcare system. Moreover, the possibility that children may have been hospitalized in other cities cannot be disregarded.

## CONCLUSIONS

High rates of admissions due to primary care-sensitive conditions, including admissions caused by acute illnesses, were observed in the present study, especially among male children. It should be noted that hospital admissions that can be prevented within primary care require longer hospital stays than those resulting from conditions that are not sensitive to primary care.
